# *QuickStats:* Percentage* of Residential Care Communities^†^ that Offer Annual Influenza Vaccination to Residents and to Employees and Contract Staff Members, by Community Bed Size — United States, 2020

**DOI:** 10.15585/mmwr.mm7140a6

**Published:** 2022-10-07

**Figure:**
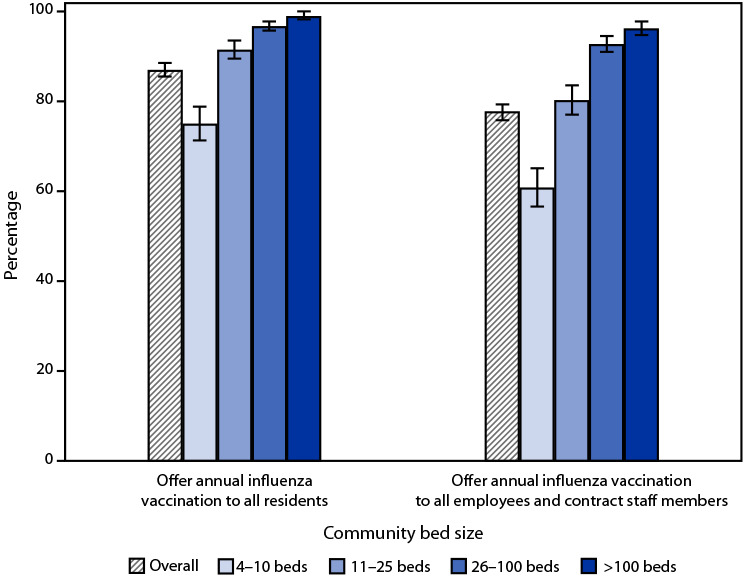
In 2020, 87.2% of residential care communities offered annual influenza vaccination to residents, and 77.8% offered annual influenza vaccination to all employees and contract staff members. The percentage of residential care communities offering annual influenza vaccination to residents and to all employees and contract staff members increased with increasing community bed size. The percentage of communities offering vaccination to residents ranged from 75.2% of communities with four to 10 beds to 91.7% with 11–25 beds, 97.0% with 26–100 beds, and 99.1% with more than 100 beds. Communities offering vaccination to all employees and contract staff members ranged from 60.9% of communities with four to 10 beds to 80.3% with 11–25 beds, 92.9% with 26–100 beds, and 96.4% with more than 100 beds.

**Source:** National Post-acute and Long-term Care Study, 2020 data. https://www.cdc.gov/nchs/npals/questionnaires.htm

